# Supplementary material for: The association between adult-life smoking and age-related cognitive decline in Danish men
Source: PLoS One. 2025 Mar 19;20(3):e0319839. doi: 10.1371/journal.pone.0319839 (PMC11922240; doi:10.1371/journal.pone.0319839)
Supplement: S1 Appendix — (DOCX) [file pone.0319839.s001.docx]

**Table S1.** Associations of adult-life smoker vs. non-smoker status with IQ change in linear regression analyses with adjustment for alcohol consumption and comorbidity index (N = 5052)*

|  | Model 1 | | | Model 2 | | | Model 3 | | |
| --- | --- | --- | --- | --- | --- | --- | --- | --- | --- |
| Predictor | Coef-  ficient | CI | P value | Coef-  Ficient | CI | P value | Coef-  ficient | CI | P value |
| Adult-life non-smoker | Ref. |  |  | Ref. |  |  | Ref. |  |  |
| Adult-life smoker | 1.89 | 1.35;2.44 | <0.001 | 1.11 | 0.60;1.62 | <0.001 | 1.24 | 0.74;1.75 | <0.001 |

^*^Model 1: adjusted for retest interval length, age at follow-up, psychiatric history, adult-life weekly alcohol consumption, years with weekly extreme binge drinking and the Charlson Comorbidity Index. Model 2 additionally adjusted for young adult IQ scores, and model 3 additionally adjusted for years of educations.

**Table S2.** Associations of adult-life smoking categorized into three pack-year categories* with IQ change in linear regression analyses with adjustment for alcohol consumption and comorbidity index (N = 3188)**

|  | Model 1 | | | Model 2 | | | Model 3 | | |
| --- | --- | --- | --- | --- | --- | --- | --- | --- | --- |
| Adult-life smokers only | Coef-  ficient | CI | P value | Coef-  ficient | CI | P value | Coef-  ficient | CI | P value |
| Light Smoker | Ref. |  |  | Ref. |  |  | Ref. |  |  |
| Moderate smoker | -0.01 | -0.78;0.76 | 0.983 | -0.67 | -1.39;0.04 | 0.066 | -0.38 | -1.10;0.33 | 0.293 |
| Heavy Smoker | -0.23 | -1.12;0.65 | 0.604 | -1.40 | -2.23;-0.57 | 0.001 | -0.96 | -1.79;-0.13 | 0.024 |

^*^Based on pack-years of smoking from age 19 to midlife follow-up

^**^Model 1: adjusted for retest interval length, age at follow-up, psychiatric history, adult-life weekly alcohol consumption, years with weekly extreme binge drinking and the Charlson Comorbidity Index. Model 2 additionally adjusted for young adult IQ scores, and model 3 additionally adjusted for years of educations.

**Table S3.** Associations of adult-life smoking categorized into three pack-year categories* with IQ change in linear regression analyses with adjustment for alcohol consumption and comorbidity index and including current smoking and smoking before age 19 (N = 3188)**

|  | Model 1 | | | Model 2 | | | Model 3 | | |
| --- | --- | --- | --- | --- | --- | --- | --- | --- | --- |
| Adult-life smokers only | Coef-  ficient | CI | P value | Coef-  ficient | CI | P value | Coef-  ficient | CI | P value |
| Light Smoker | Ref. |  |  | Ref. |  |  | Ref. |  |  |
| Moderate smoker | -0.04 | -0.81;0.74 | 0.927 | -0.63 | -1.35;0.10 | 0.089 | -0.35 | -1.08; 0.37 | 0.338 |
| Heavy Smoker | -0.03 | -0.96;0.89 | 0.945 | -1.10 | -1.97;-0.24 | 0.012 | -0.68 | -1.55;0.18 | 0.122 |
| Current smoking | -1.79 | -2.59;-0.99 | <0.001 | -1.72 | -2.47;-0.98 | <0.001 | -1.69 | -2.43;-0.95 | <0.001 |
| Smoking before age 19 | 2.09 | 1.32; 2.86 | <0.001 | 1.51 | 0.80;2.23 | <0.001 | 1.69 | 0.98;2.40 | <0.001 |

^*^Based on pack-years of smoking from age 19 to midlife follow-up

^**^Model 1: adjusted for retest interval length, age at follow-up, psychiatric history, adult-life weekly alcohol consumption, years with weekly extreme binge drinking, and the Charlson Comorbidity Index. Model 2 additionally adjusted for young adult IQ scores, and model 3 additionally adjusted for years of educations.

**Table S4.** Associations of adult-life smoking analyzed as a continuous pack-year variable* with IQ change in linear regression analyses (N = 3188 respectively)**

|  | Model 1 | | | Model 2 | | | Model 3 | | |
| --- | --- | --- | --- | --- | --- | --- | --- | --- | --- |
| Predictor | Coef-  ficient | CI | P value | Coef-  ficient | CI | P value | Coef-  ficient | CI | P value |
| Pack-years linear | -0.02 | -0.04;0.01 | 0.161 | -0.05 | -0.07;-0.03 | <0.001 | -0.36 | -0.06;-0.01 | 0.002 |
| Pack-years  Quadratic | 1.2*10^-4^ | -3.4*10^-5^;  2.7*10^-4^ | 0.126 | 2.1*10^-4^ | 7.0*10^-5^;  3.6*10^-4^ | 0.004 | 1.6*10^-4^ | 1.4*10^-5^;  3.0*10^-4^ | 0.031 |
|  | | | | | | | | | |
| Model*** | Coef-  ficient | CI | P value | Coef-  ficient | CI | P value | Coef-  ficient | CI | P value |
| Pack-years linear | -0.01 | -0.04;0.01 | 0.342 | -0.04 | -0.05;-0.01 | 0.001 | -0.03 | -0.05;-0.01 | 0.019 |
| Pack-years quadratic | 1.1*10^-4^ | -5.2*10^-5^;  2.6*10^-4^ | 0.188 | 1.9*10^-4^ | 4.0*10^-5^;  3.3*10^-4^ | 0.013 | 1.3*10^-4^ | -1.2*10^-5^;  2.8*10^-4^ | 0.072 |
| Current smoking | -1.85 | -2.65;-1.05 | <0.001 | -1.76 | -2.51;-1.02 | <0.001 | -1.73 | -2.47;-0.99 | <0.001 |
| Smoking before age 19 | 2.08 | 1.31;2.85 | <0.001 | 1.53 | 0.81;2.24 | <0.001 | 1.70 | 0.99;-2.42 | <0.001 |

^*^Based on pack-years of smoking from age 19 to midlife follow-up

^**^Model 1: adjusted for retest interval length, age at follow-up, and psychiatric history. Model 2 additionally adjusted for young adult IQ scores, and model 3 additionally adjusted for years of educations.

***Models additionally included current smoking and snoking before age 19.
